# 698. Contemporary Clinical Epidemiology of Pediatric *Shigella* and *Campylobacter* Infections in Houston, TX, 2019 and 2020

**DOI:** 10.1093/ofid/ofab466.895

**Published:** 2021-12-04

**Authors:** christy tabarani, Anthony R Flores, Anthony R Flores, Cesar A Arias, Audrey Wanger

**Affiliations:** 1 University of Texas, McGovern Medical School, Houston, TX; 2 McGovern Medical School, Houston, TX; 3 CARMiG, UTHealth and Center for Infectious Diseases, UTHealth School of Public Health, Houston, TX; Molecular Genetics and Antimicrobial Resistance Unit and International Center for Microbial Genomics, Universidad El Bosque, BOG, COL, Houston, Texas; 4 University of Texas Health Science Center, University of Texas Health Science Center, Houston, TX

## Abstract

**Background:**

Infections due to Gram-negative, diarrheal pathogens are a significant cause of morbidity in children. Clinical features of pediatric *Shigella* and *Campylobacter* infections in urban cities in the United States are not well described.

**Methods:**

We used a retrospective chart review of records (0-18 years of age) from a network of hospitals in Houston, TX. Only patients with *Shigella* spp. or *Campylobacter* spp. isolated from clinical samples in 2019 and 2020 were included. Demographic, clinical, and microbiological data were extracted from the medical record.

**Results:**

We identified a total of 59 and 16 pediatric patients with *Shigella* spp. and *Campylobacter* spp. infections, respectively. Hospital admission occurred in 27.1% (16/59) of *Shigella* and 25% (4/16) of *Campylobacter.* Length of stay ranged between 1 and 2 days for both pathogens (Table 1). Of cases with available clinical data, *Shigella* infections were more likely to report fever during their illness compared to *Campylobacter* (80% versus 45.4%) (Table 2). Seizures were observed in 4 *Shigella* infected patients. No episodes of *Shigella* or *Campylobacter* bacteremia were identified. Among patients with an identified exposure, daycare attendance and contact with individuals experiencing similar symptoms were most common (Table 2). The vast majority of *Shigella* species were *S. sonnei* (96.6%) and all *Campylobacter* were *C. jejuni* (Table 3). Resistance to trimethoprim-sulfamethoxazole (TMP-SMX) was common (40/55, 72.7%) among *Shigella* isolates tested. No resistance to fluoroquinolones or third generation cephalosporins in any of the *Shigella* spp. isolates was observed. Susceptibility testing was not performed in *Campylobacter* due to lack of isolates. The most frequent antibiotic used was azithromycin (in 73.3% and 75% of patients with *Shigella* and *Campylobacter,* respectively). Major complications included urinary tract infection (n=1), rectal prolapse (n=1) and splenomegaly (n=1).

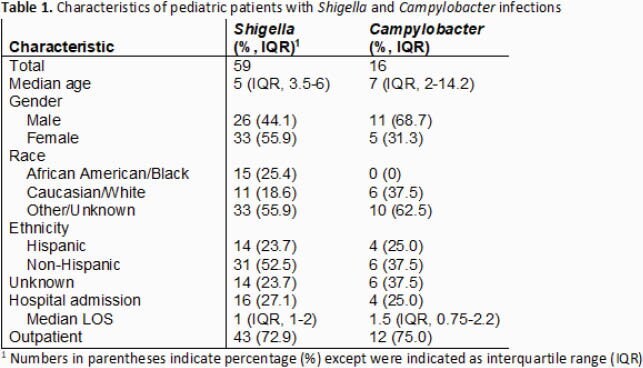

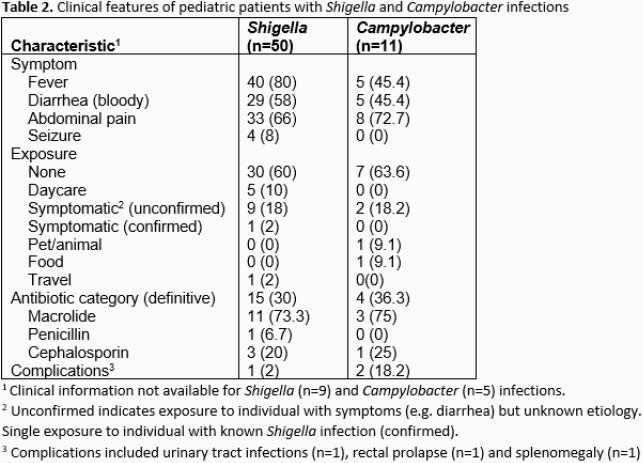

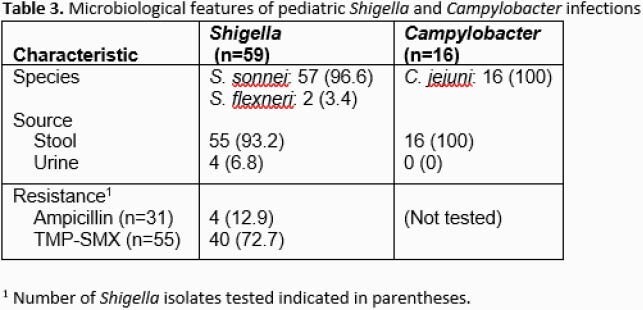

**Conclusion:**

Infections due to *Shigella* and *Campylobacter* were a significant burden among pediatric patients between 2019 and 2020 in Houston, TX. The observed high frequency of resistance to TMP-SMX and emergence of multi-drug resistant *Shigella* in other countries warrants continued surveillance.

**Disclosures:**

**Anthony R. Flores, MD, MPH, PhD**, Nothing to disclose **Cesar A. Arias, M.D., MSc, Ph.D., FIDSA**, **Entasis Therapeutics** (Grant/Research Support)**MeMed Diagnostics** (Grant/Research Support)**Merk** (Grant/Research Support)

